# Inflammatory Bowel Disease Therapies and Gut Function in a Colitis Mouse Model

**DOI:** 10.1155/2013/909613

**Published:** 2013-08-06

**Authors:** Lily Nahidi, Steven T. Leach, Hazel M. Mitchell, Nadeem O. Kaakoush, Daniel A. Lemberg, John S. Munday, Karina Huinao, Andrew S. Day

**Affiliations:** ^1^School of Women's and Children's Health, University of New South Wales, Randwick, Sydney, NSW 2031, Australia; ^2^School of Biotechnology and Biomolecular Sciences, University of New South Wales, Randwick, Sydney, NSW 2052, Australia; ^3^Department of Gastroenterology, Sydney Children's Hospital, Randwick, Sydney, NSW 2031, Australia; ^4^Department of Pathology, Institute of Veterinary, Animal and Biomedical Sciences, Massey University, Palmerston North 4442, New Zealand; ^5^Paediatric Gastroenterology, Christchurch Hospital, Christchurch 8140, New Zealand; ^6^Department of Paediatrics, University of Otago, Christchurch, Christchurch 8140, New Zealand

## Abstract

*Background*. Exclusive enteral nutrition (EEN) is a well-established approach to the management of Crohn's disease. *Aim*. To determine effects of EEN upon inflammation and gut barrier function in a colitis mouse model. *Methods*. Interleukin-10-deficient mice (IL-10^−/−^) were inoculated with *Helicobacter trogontum* and then treated with EEN, metronidazole, hydrocortisone, or EEN and metronidazole combination. Blood and tissue were collected at 2 and 4 weeks with histology, mucosal integrity, tight junction integrity, inflammation, and *H. trogontum* load evaluated. *Results*. *H. trogontum* induced colitis in IL-10^−/−^ mice with histological changes in the cecum and colon. Elevated mucosal IL-8 mRNA in infected mice was associated with intestinal barrier dysfunction indicated by decreased transepithelial electrical resistance and mRNA of tight junction proteins and increased short-circuit current, myosin light chain kinase mRNA, paracellular permeability, and tumor necrosis factor-**α** and myeloperoxidase plasma levels (*P* < 0.01 for all comparisons). EEN and metronidazole, but not hydrocortisone, treatments restored barrier function, maintained gut barrier integrity, and reversed inflammatory changes along with reduction of *H. trogontum* load (versus infected controls *P* < 0.05). *Conclusion*. *H. trogontum* infection in IL-10^−/−^ mice induced typhlocolitis with intestinal barrier dysfunction. EEN and metronidazole, but not hydrocortisone, modulate barrier dysfunction and reversal of inflammatory changes.

## 1. Introduction 

Inflammatory bowel diseases (IBDs), including ulcerative colitis (UC) and Crohn's disease (CD), are a group of conditions characterized by chronic relapsing inflammation of the gastrointestinal tract [1–4]. Despite extensive research conducted over many years, the causes of IBD are still unclear. However, a defective intestinal epithelial barrier and continuous bacterial antigen stimulation of mucosal immunity have been proposed as important etiological factors of IBD in genetically susceptible individuals [5–7].

It is well accepted that luminal bacteria play an important role in the initiation and progression of IBD [8, 9]. The strongest evidence for this conclusion comes from studies utilizing rodent models of colitis [8, 10] and is also supported by clinical observations [5, 6]. A number of animal studies have employed the interleukin-10 deficient mice (IL-10^−/−^) mouse model, given that IL-10 is known to suppresses the secretion of numerous proinflammatory cytokines [11, 12]. This model has been shown to readily develop moderate-to-severe IBD when triggered by events that compromise their mucosal barrier, such as *Helicobacter* species [8]. While many studies using the IL-10^−/−^ *Helicobacter* model have focused on *H. hepaticus *or *H. bilis*, other enterohepatic species including *H. cinaedi*, *H. typhlonius*, *and H. trogontum* (a Gram-negative microaerophilic bacterium initially isolated from rats) [13, 14] have also been shown to initiate colitis in IL-10^−/−^ mice [10, 15, 16]. For instance, in the study of Whary et al., in which both *Helicobacter* free IL-10^−/−^ (B6 background) and B6 mice were infected with *H. trogontum*, a rapid development of an acute-on-chronic typhlocolitis, epithelial hyperplasia, and dysplasia was observed following infection of *Helicobacter *free IL-10^−/−^ mice on a B6 background with *H. trogontum* [10]. Based on these findings, Whary and colleagues stated that “this model should prove useful in dissecting the pathogenesis of various clinical and pathological features noted in inflammatory bowel disease (both CD and UC) of humans.” The choice of this species is further supported by a recent study by our group that has demonstrated that *H. trogontum* can adhere to host cells through flagella-microvillus interactions and invade causing a membrane ruffling-like effect and severe cell damage, which may account for loss of barrier function following infection with this bacterium [17]. Further, in the same study we identified within the secretome fraction of *H. trogontum* three proteins belonging to the type six secretion system, which we also showed were present in *H. bilis*, *H. hepaticus,* and *H. cinaedi* [17]. We have also detected *H. trogontum* DNA in biopsy and fecal samples of children with CD [18, 19].

It is generally accepted that luminal antigen and adjuvants from intestinal microbiota drive an inflammatory response in the intestinal mucosa. Tumor necrosis factor (TNF)-*α* is a key proinflammatory mediator in this mucosal response and subsequently stimulates additonal cytokines including IL-8 and myeloperoxidase (MPO) which promote inflamamtory cell recruitment and activation. This mucosal response causes disruption of intestinal tight junction barrier function resulting in an increase in permeability to additional luminal antigen and adjuvants, which further drives the response [20, 21]. Given this, mechanisms that control barrier function and the action of TNF-*α* and other proinflammatory effectors are extremely important in maintaining disease control as well as intestinal tissue integrity.

Several therapeutic approaches are used for the treatment of IBD including antibiotics, corticosteroids, and biological agents. Previously, the goal of therapy was to reduce inflammation, and although this is still a priority, achieving mucosal healing is now considered the gold standard of therapy. Unfortunately, while current therapies can rapidly alleviate symptoms, they do not cure IBD and are limited with improvements in mucosal inflammatory lesions and mucosal healing [22–24]. The only exception to this are biological therapies, which can robustly control mucosal inflammation and induce mucosal healing along with improvement of clinical symptoms [25–27]. However, similar to antibiotics and corticosteroids, they are associated with undesirable side effects consequent to prolonged use [14, 22–24, 28, 29]. 

The rationale for antibiotic treatment, including metronidazole (MNZ), ornidazole, and ciprofloxacin, in IBD is based upon a body of evidence showing that luminal bacteria have a role in the pathogenesis of IBD [24, 30]. These antibiotics are used in clinical practice for controlling symptoms and preventing septic complications in patients with active CD [24, 31]. They are known to alter the bacterial composition of the intestine by changing the balance between different bacterial groups [32, 33]. This may explain their role in treating the exacerbation of CD in clinical practice [34–36]. In chronic inflammatory diseases antibiotics can directly or indirectly suppress the intestine's immune system to reduce disease severity [24, 34–36].

The rationale for the use of corticosteroids in IBD relates to their efficacy in current clinical practice to achieve rapid symptomatic relief in many patients [37, 38]. Two commonly used corticosteroids are hydrocortisone (HC) and methylprednisolone. Despite the dramatic success of these corticosteroid medications in achieving clinical remission in CD patients, they have not been shown to have an impact on the maintenance of long-term remission or upon mucosal healing [37].

Over the last 3 decades exclusive enteral nutrition (EEN) has been demonstrated not only to be a valid therapeutic option for induction and maintenance of remission of CD in both adults and children but also to have very few side effects [28, 39, 40]. EEN involves the administration of a liquid elemental or polymeric formula (PF) exclusively, with cessation of normal diet for a period of 6 to 8 weeks [22, 28]. Elemental formula contains individual amino acids, while PF is composed of intact proteins. Pediatric studies have demonstrated that PF not only has equivalent efficacy to elemental formula, but due to its improved palatability, it is also associated with increased tolerance and compliance [22, 41]. PF, as used for EEN, improves nutritional status and can induce clinical remission [22, 28, 39]. A number of studies have reported that clinical and endoscopic improvements in pediatric CD are associated with reduced mucosal mRNA levels of key proinflammatory mediators [22, 39, 42–44]. 

Despite the well-defined clinical benefits of EEN [22, 39, 44], its mechanisms of action remain unclear. Our aim was to use a mouse model of colitis to investigate the mucosal healing associated with various treatments of IBD. We investigated 4 first-line CD therapies [EEN, HC (in an oral liquid form), MNZ, and a combination of EEN and MNZ], which are used at diagnosis at the Sydney Children's Hospital, Australia. These therapies were chosen to replicate the therapeutic aspects of human CD in IL-10^−/−^ murine model of colitis. To study mucosal healing, we measured transepithelial electrical resistance (TEER: a measure of tight junction integrity), short-circuit current (Isc: a measure of charged ion flow across a membrane), the tight junction associated proteins including claudin-1, occludin and zonula occludens (ZO)-1, and myosin light chain kinase (MLCK) which have been shown to be accurate and reliable measures of intestinal barrier function [45–51].

## 2. Materials and Methods

### 2.1. Reagents

PCR primers and TRIzol reagent were purchased from Invitrogen (Carlsbad, CA, USA). MNZ, HC, and basic chemicals were obtained from Sigma Chemical Company (St. Louis, MO, USA). Osmolite (Abbott Nutrition, Australia Pty Ltd, Sydney, NSW; the composition of Osmolite is provided in Supplementary Table 1 of the Supplementary Material available online at http://dx.doi.org/10.1155/2013/909613) was used for PF.

### 2.2. Preparation of *H. trogontum* Inoculation

*H. trogontum* LRB 8581 (susceptible to MNZ) was grown on Horse Blood Agar [Blood Agar Base no. 2 supplemented with 8% defibrinated horse blood (Oxoid; Heidelberg West, VIC, Australia)], and incubated under microaerobic conditions for 72 hours at 37°C. The cells were then harvested from the plates using 0.9% saline solution, washed, and then transferred to fresh 0.9% saline solution to obtain an inoculum of 1 × 10^8^ CFU/mL.

### 2.3. Model of Colitis in IL-10^−/−^ Mice on a C57BL/6 Background

Six-to-eight-week-old IL-10^−/−^ mice on a C57BL/6 background were bred at the Australian Bioresources Centre, Moss Vale, Australia. Given that the presence of *Helicobacter* species in the gastrointestinal tract of mice has been associated with spontaneous colitis development, the IL-10^−/−^ mice used in this study were bred and maintained in conditions that ensured they were *Helicobacter* free prior to the commencement of the study. All mice (*n* = 71; Table 1) were housed in microisolator cages in groups of 2–6, in a 12-hour light/dark cycle and a constant temperature of 20 ± 5°C. All experiments were approved by the Animal Care and Ethics Committee (ACEC) of the University of New South Wales, Sydney, Australia (no. 11/15B). 

For induction of colitis, each mouse was orogastrically inoculated with 100 *μ*L of *H. trogontum* on 3 occasions over the period of 5 days. Immediately after the final inoculation, clinically relevant treatments were commenced as described in Table 1. All treatment regimens were given in a liquid form in the drinking bottle *ad libitum*. The liquids were replaced every 24 hours. The concentrations of HC and MNZ provided in the drinking bottle were calculated based on the average liquid intake and average weight of each animal. The average mouse weight at 8 weeks was approximately 25 g, with an average daily water consumption of 4 mL. Based on these values, the following concentrations, extrapolated from human doses, were used: HC at a dose of 5 mg/kg/day [52]; MNZ at a dose of 10 mg/kg/day [32]. PF was provided as a sole nutrient/water source [22, 28] with exclusion of standard mouse chow (Lab Diet 5001; PMI Nutrition Inc., LLC, Brentwood, MO; the chemical composition of the standard mouse chow diet is provided in Supplementary Table 2).

Over the period of the study, the mice were monitored daily for diarrhea and any signs of dehydration (loss of skin elasticity) shallow breathing, unkempt fur, and extraordinary/antisocial behavior, and the body weight of the mice was measured every 48 hours. If any signs of severe diarrhea, dehydration, and weight loss (greater than 20% of original body weight) developed, they were euthanized immediately. 

Two weeks after initiation of the treatments, half the animals were euthanized and tissue was collected (*n* = 33). The remaining animals (*n* = 29) were euthanized after 4 weeks and tissue collected. All the animals in the HC group were euthanized when moribund at week 3 rather than week 4 due to ethical considerations. However, their indexes were compared to those of other groups at week 4 throughout this study. At necroscopy, blood was collected by cardiac puncture with cecum and colon also collected and fixed in 10% formalin for histological injury evaluation and immunofluorescence labelling. Colon was also collected into *RNAlater* [40 mL 0.5 M EDTA (Sigma), 25 mL 1.0 M sodium citrate (Sigma), 700 g ammonium sulfate (Sigma), and 935 mL distilled water at pH of 5.2] and stored at −20°C for PCR examinations, and fresh tissue was used for assessment of mucosal integrity and permeability in an Ussing chamber. 

### 2.4. Histological Examination

Macroscopic evaluation was based on criteria reflecting inflammation, including bowel wall thickening, luminal distension and bleeding, intestinal tissue necrosis and rupture, intestinal edema, and erythema and haemorrhage. Following macroscopic evaluation, cecum and colonic tissues were fixed in 10% buffered formalin solution and embedded in paraffin. The embedded specimens were then sectioned (6 *μ*m), stained with Hematoxylin and Eosin, and examined by light microscopy. The slides were assigned a histological injury score using a scheme modified from Madsen et al. [9]. This score ranged from 0 (minimal injury) to 15 (maximal injury) by scoring 4 criteria [9], epithelial hyperplasia (0–3), lamina propria mononuclear and neutrophilic infiltration (0–4 and 0–4, resp.), and mucosal ulceration (0–4; Supplementary Table 3). A veterinary pathologist who was blind to the treatment received carried out the histological scoring. The mucosal thickness was also measured. This was undertaken by taking a photomicrograph of each section and using image analysis software to measure the thickness.

### 2.5. Ussing Chamber Experiments

The Ussing chamber (World Precision Instruments Inc., FL, USA) was used to measure the TEER and Isc of fresh colonic tissues. Colon segments were immediately excised postmortem and placed in a fresh Ussing chamber buffer (116.0 mM NaCl, 5.4 nM KCl, 0.4 mM MgCl_2_, 1.8 mM CaCl_2_, 5.5 mM glucose, 26.0 mM NaHCO_3_, and 0.9 mM NaH_2_PO_4_ at pH of 7.4). Tissue segments were then opened along the mesenteric border, cut into a flat sheet, and rinsed free of luminal contents with phosphate buffered saline (PBS; Gibco Invitrogen, Carlsbad, CA., USA) prior to being vertically mounted in the Ussing chamber. The chamber exposed tissues to 10 mL of circulating oxygenated buffer at 37°C on both the mucosal and serosal sides. The serosal bathing solution contained 10.0 mM glucose as a source of energy, which was osmotically balanced by mannitol (10.0 mM) on the mucosal side. The chamber was connected to matched voltage and current electrodes (EKV and EKC, resp.; World Precision Instruments Inc.) through a KCl saturated agar bridge to monitor the potential differences across the tissues. Following equilibration for 30 minutes, the Isc, an index of net active ion passage, was recorded continually in the voltage clamp mode at 0 potential difference. A voltage pulse of 2 mV was imposed for 3 seconds at 60-second intervals across the colon tissues in order to estimate the TEER (expressed as Ω·cm^2^), as a surface area normalized ratio of imposed voltage pulse to the observed deflection in resultant circuit current, according to the Ohm's law formula:

(1) TEER  (an  index  of  the  tissue  viability  and  integrity  over  time)=(ΔVΔI)A  (A  is  an  exposed  window  surface  area  of  1 cm2).

### 2.6. Permeation Experiments

In addition to electrophysiological measurements, the mucosal-to-serosal permeability of fresh colonic tissues based on a paracellular marker was assessed using the Ussing chamber. Type VI horseradish peroxidase (HRP; Sigma), with a molecular mass of ~44 kDa, was used as a model protein probe. HRP is an established paracellular marker and has been extensively utilized to study macromolecular permeability [45, 47–49, 53]. As it is a relatively large molecule (similar in size to antigenic proteins known to stimulate immune responses), it cannot readily diffuse across the cell membrane nor pass through intact tight junctions making it a good candidate to measure tight junctional impairments [45, 49]. Further, the amount of HRP crossing the epithelial layer can be easily measured using a kinetic enzymatic assay [47–49, 53]. In the permeation experiments, HRP (10.0^−5 ^M) [45, 47–49] was added to the mucosal buffer once equilibrium was reached (30 minutes after colon tissues had been placed in the Ussing chamber). After 1 minute, serosal samples (500 *μ*L) were collected, after which they were collected every 30 minutes for 2 hours. On each occasion the collected buffer was replaced with fresh buffer to maintain constant volume. Enzymatic activity of HRP was measured using a modified kinetic assay as previously described [45].

### 2.7. Enzyme-Linked Immunosorbent Assay (ELISA) for TNF-*α* and MPO

To measure the plasma levels of TNF-*α* (a master proinflammatory cytokine of the innate immune response) and MPO (a marker of eosinophil and neutrophil activation) [54, 55], TNF-*α* (abcam, CA, USA) and MPO (abcam) ELISAs were performed using commercial kits according to the manufacturers' instructions. The minimum detectable dose of TNF-*α* was less than 60 pg/mL, while that of MPO was 0.6 ng/mL. The absorptions were measured at 450 nm in an ELISA microplate reader (BioTek, NY, USA). The results are expressed as percentage of the noninfected controls over the period of 4 weeks.

### 2.8. RNA Extraction and Real-Time (RT) PCR

Total RNA was extracted from tissue which had been stored in *RNAlater* by manually homogenizing the tissues with a mortar and pestle in TRIzol reagent (Invitrogen). To remove contaminating DNA, the Turbo DNA free kit (Ambion, Austin, USA) was utilized as described previously [45]. The total RNA concentration was determined by reading the absorbance at 260/280 nm using a NanoDrop ND-1000 spectrophotometer (NanoDrop Technologies, Wilmington, DE, USA). cDNA was then synthesized through reverse transcription reaction using superscript III reverse transcriptase enzyme (Invitrogen) according to the manufacturer's instruction. Reactions were carried out in a thermocycler (Corbett Research, Australia).

RT-PCRs were carried out using the Realplex Mastercycler (Eppendorf, Barkhausenweg, Hamburg, Germany) with the SYBR-green (BioRad) fluorescence quantification system as previously described [45]. In a total volume of 25 *μ*L, each RT-PCR reaction contained primer pair mix (Invitrogen), 12.5 *μ*L of 1x iQ TM SYBR Green RT-PCR buffer, nuclease-free water (Ambion), and 5 *μ*L of cDNA (2 ng/*μ*L) as a template, except for negative controls. All the RT-PCR primers were specifically designed for mouse occludin, claudin-1, ZO-1, myosin light chain kinase (MLCK), macrophage inflammatory protein 2 (MIP-2), lipopolysaccharide-induced CXC chemokine (LIX), TNF-*α* and *β*2-microglobulin (*β*2M) as a reference. Primer sequences for the RT-PCR were as follows: (1) mouse occludin [56], forward: 5′-ATG TCC GGC CGA TGC TCT C-3′, reverse: 5′-CTT TGG CTG CTC TTG GGT CTG TAT-3′; (2) mouse claudin-1 [57], forward: 5′-AGG AAA GGC CCT TCA GCA GAG CAA-3′, reverse: 5′-GTG CCC CCT CTT GACT CAT GCA AC-3′; (3) mouse ZO-1 [56], forward: 5′-ACC CGA AAC TGA TGC TGT GGA TAG-3′, reverse: 5′-AAA TGG CCG GGC AGA GAC TTG TGT A-3′; (4) mouse MLCK [58], forward: 5′-ACA TGC TAC TGA GTG GCC TCT CT-3′, reverse: 5′-GGC AGA CAG GAC ATT GTT TAA GG-3′; (5) mouse MIP-2 [59], forward: 5′-CGC CCA GAC AGA AGT CAT AG-3′, reverse: 5′-TCC TCC TTT CCA GGT CAG TTA-3′; (6) mouse LIX [59], forward: 5′-GGT CCA CAG TGC CCT ACG-3′, reverse: 5′-GCG AGT GCA TTC CGC TTA-3′; and (7) mouse TNF-*α* [60], forward: 5′-ATC ATC TTC TCA AAA TTC GAG TGA C-3′, reverse: 5′-CTA GTT GGT TGT CTT TGA GAT CCA T-3′. The cycle profile consisted of denaturation at 95°C for 2 minutes, followed by 40 cycles of 95°C for 20 seconds, 63°C for 30 seconds, and 72°C for 60 seconds. The PCR products were then analyzed for homogeneity by melting curve analysis. No primer dimers were detected during the 40 amplification cycles. Realplex software was used to calculate the cycle threshold (Ct) for each reaction. The results were expressed as 2^−ΔΔ^ Ct. Reported Ct value for each sample is an average of the values obtained from 4 independent runs. 

### 2.9. Detection of *H. trogontum* in the Colon of Mice Using 16S rRNA PCR

DNA was extracted from approximately 200 mg of colonic tissue, which had been stored in *RNAlater*, using the Qiagen Puregene Core kit A (Qiagen, Hilden, Germany) according to the manufacturer's instructions. Although we were able to determine *H. trogontum* colonization status by PCR in the majority of the mice, in some mice insufficient colonic tissue was available. However, as histological assessment, TEER, Isc, HRP flux, gene expression of MIP-2, LIX, and TNF-*α* were determined in colonic samples from all mice, colonization status was inferred based on these parameters.

The concentration and quality of DNA were measured using a NanoDrop ND-1000 Spectrophotometer (NanoDrop Technologies). The *H. trogontum* PCR was performed using the primer pair B72 and B39 previously designed by Mendes et al. [13]. The PCR reactions were performed in a 25 *μ*L reaction mixture consisting of 10 pmol of each primers B72 (forward: 5′-CATAGGTAACATGCCCCA-3′; Sigma) and B39 (reverse: 5′-CTGTTTTCAAGCTCCCC-3′; Sigma), 1x PCR buffer (Fisher Biotech, Wembley, WA, Australia), 200.0 *μ*M of each deoxynucleoside triphosphate (dNTP; Fisher Biotech), 2.0 mM MgCl_2_ (Fisher Biotech), 0.825 U of Taq polymerase (Fisher Biotech), and 2 *μ*L of DNA. The thermal cycling conditions for the primers B79 and B39 were 94°C for 45 seconds, 63°C for 45 seconds, and 72°C for 1 minute for 25 cycles. Following the PCR, 5 *μ*L of the PCR products was subjected to gel electrophoresis (with a constant voltage of 100 for 22 minutes), stained with 1X GelRed Nucleic Acid Gel Stain (Biotium, Hayward, CA, USA), and visualized under UV transillumination (BioRad, Hercules, USA). For PCR analysis, samples resulting in 888 bp fragment and displaying a single band were considered PCR positive for *H. trogontum*. The limit of detection of the PCR was <40 pg DNA.

### 2.10. Immunofluorescence Staining of ZO-1 Protein

Paraffin-embedded colonic sections (6 *μ*m) were used for fluorescent labelling of the tight junction protein ZO-1. Paraffin-embedded sections were dewaxed in Histoclear (National Diagnostics, Atlanta, GA, US) for 10 minutes before rehydrating with 100%, 75%, and 50% ethanol (Fronine laboratory supplies, Australia). Prior to application of primary antibody, antigen retrieval was performed by heating the sections in Tri-sodium citrate buffer [1.47 g Tri-sodium citrate (Sigma), 2.2 mL 1.0 M HCl (Sigma), and 500 mL distilled water at pH 6.0] for 30 seconds on high power in microwave (1000 W). Sections were then rinsed 3 times (for 5 minutes) with PBS (Gibco) blocked with 10% normal donkey serum (Sigma) in 0.1% bovine serum albumin (Sigma) in PBS for 20 minutes at room temperature. Sections were then incubated for a further hour with primary antibody mouse anti-ZO-1 (1 : 200 diluted in 2% normal donkey serum; Invitrogen). Following 3 washes in PBS, the slides were incubated with donkey anti-mouse ALEXA 488 (1 : 500 diluted in 2% normal donkey serum; Invitrogen) at room temperature, for 30 minutes in the dark. After 3 more washes with PBS, slides were mounted with Vectashield + DAPI mounting medium (Vector laboratories, Burlingame, CA, USA) and were imaged using an Axioplan 2 microscope (Zeiss, Oberkochen, Germany) and AxioVision software (Zeiss).

### 2.11. Statistical Analysis

Data are presented as mean ± standard deviation (SD) and analyzed using GraphPad Prism software (version 4.0 for windows; GraphPad Software, San Diego, CA, USA). Statistical analysis employed the one-way ANOVA test for comparison of multiple groups with a *Tukey's* posttest to assess statistical differences among all groups. Spearman's correlation was used for all correlations. The bacterial qualitative comparisons were performed using the Fisher exact test. *P* values of ≤0.05 were considered to be statistically significant. 

## 3. Results

### 3.1. Body Weight

**Figure 1:**
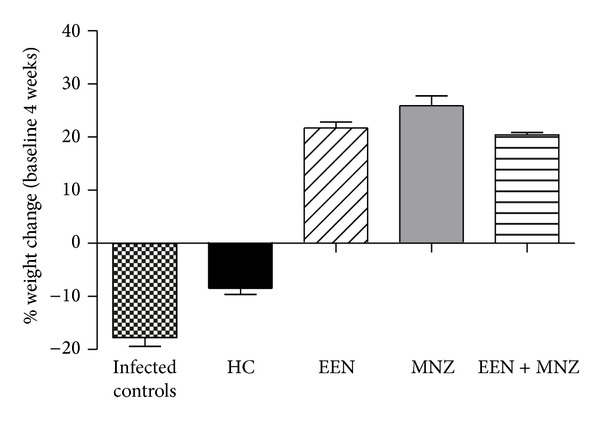
Average body weight of male and female C57BL/6 mice. Six-to-eight-week-old IL-10^−/−^ mice (male and female) were infected with 100 *μ*L 1 × 10^8^ CFU/mL of *H. trogontum* and then treated with hydrocortisone (HC), exclusive enteral nutrition (EEN), metronidazole (MNZ), or a combination of EEN and MNZ commenced. In the infected control groups, *H. trogontum* caused a significant weight loss over the period of 4 weeks (versus noninfected controls *P* < 0.01). However, infected but (EEN, MNZ, and EEN + MNZ) treated mice gained weight over the 4-week treatment period (versus infected controls *P* < 0.01 for all comparisons). Mice in the HC group rapidly developed weight loss over the 3-week experimental period (versus infected controls *P* = NS; versus noninfected controls *P* < 0.01). NS: not significant; *P* > 0.05.

Infected untreated mice (infected controls) showed moderate-to-severe weight loss over the period of 4 weeks (versus noninfected controls *P* < 0.01; Figure 1). Similarly, infected mice treated with HC showed weight loss over the duration of 3 weeks (versus infected controls *P* = NS; Figure 1). However, infected mice treated with EEN, MNZ, or EEN + MNZ gained weight over the 4-week experimental period (versus infected controls *P* < 0.01 for all comparisons; Figure 1).

### 3.2. *H. trogontum* Infection and Association with Onset of Typhlocolitis in Mice

All mice, except for the noninfected controls, were infected with *H. trogontum* by 3 inoculations over 5 days. The presence of *H. trogontum* in the colon was determined at 2 weeks and 4 weeks using an *H. trogontum* 16S rRNA specific PCR. 

*H. trogontum* DNA was not detected in any of the assayed colon specimens from noninfected controls (*n* = 4; 0% positive; Supplementary Table 4 and Supplementary Figure 1). At 2 and 4 weeks, *H. trogontum* DNA was detected in 75% of colon specimens collected from infected controls (Supplementary Table 4 and Supplementary Figure 1). In contrast, *H. trogontum* DNA was not detected in any of the colon specimens obtained from infected EEN treated mice (weeks 2 and 4: 0% positive; Supplementary Table 4 and Supplementary Figure 1). In the MNZ and the EEN + MNZ treated mice, *H. trogontum* was detected in only 25% of the mice (Supplementary Table 4 and Supplementary Figure 1). *H. trogontum* was detected in 100% of the colon specimens collected from the infected but HC treated mice at the 2- and 3-week time points (Supplementary Table 4 and Supplementary Figure 1). 

Although *H. trogontum* could only be detected in 75% of the mice infected with *H. trogontum* (Supplementary Table 4 and Supplementary Figure 1), as shown later, all of the mice in this group were shown to have a moderate-to-severe typhlocolitis with elevated levels of inflammatory markers (at 2 and 4 weeks), which would strongly suggest that infection was present in these mice. The failure to detect* H. trogontum* was likely due to the fact that the number of *H. trogontum* in the colonic samples collected fell below the limit of detection of the PCR (<40 pg DNA). 

**Figure 2:**
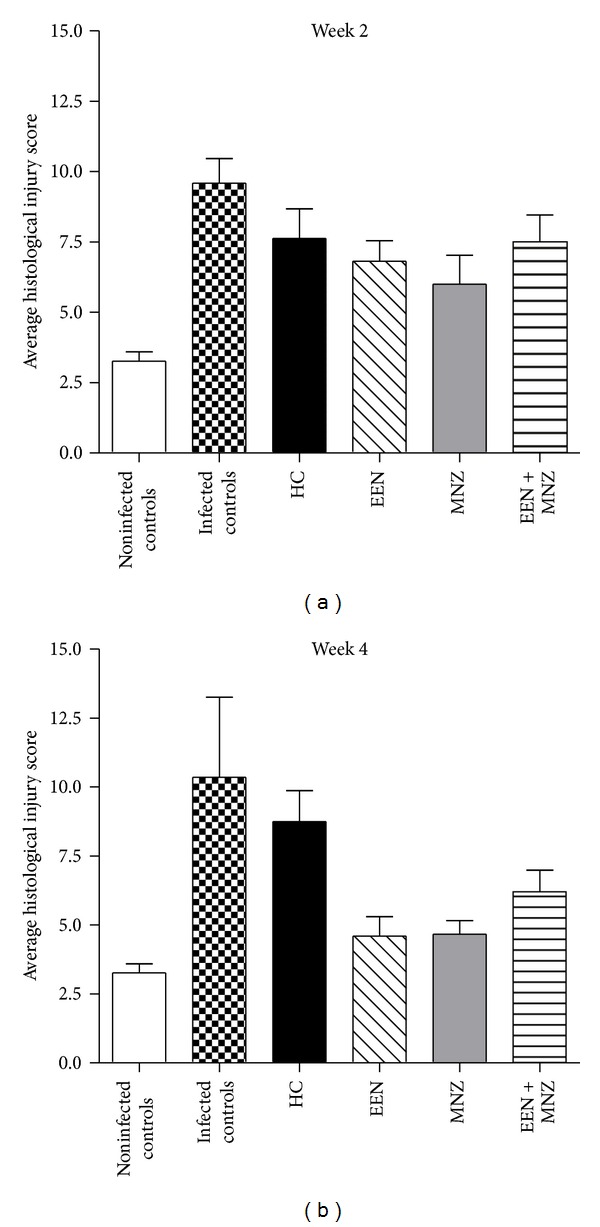
Histopathological changes in the colon of noninfected and infected/treated mice. Hematoxylin and Eosin stained sections of the colon from each mouse were examined by light microscopy at week 2 (a) and week 4 (or week 3 for HC group) (b) after the commencement of treatment. Mucosal ulceration, epithelial hyperplasia, lamina propria mononuclear, and neutrophil infiltration were scored on an ascending scale, from 0 to 15, of severity of histological injury. In the infected animals, the total colonic histological injury score was significantly higher than that of the noninfected controls (weeks 2 and 4: *P* < 0.01 for both comparisons). The histological injury score was significantly less in infected groups that had undergone 2–4 weeks of treatments (weeks 2 and 4: versus infected controls *P* < 0.01 for all comparisons). The only exception to this was with HC, where a slight decrease in colonic histological injury score was noted; however, the differences were not significant (week 2 and 3: versus infected controls *P* = NS for both comparisons; versus noninfected controls *P* < 0.01 for both comparisons). NS: not significant; *P* > 0.05. HC: hydrocortisone; EEN: exclusive enteral nutrition; MNZ: metronidazole.

The macroscopic appearance of the cecum and colon of noninfected control mice was normal as was the histological appearance, as indicated by minimal/no mononuclear or polymorphonuclear cell infiltration, minimal/no evidence of histological injury, erosion or epithelial hyperplasia. The average histological injury score for the noninfected controls was 3 ± 3 for cecum and 3 ± 1 for colon (Figures 2(a) and 2(b)). In contrast, in the *H. trogontum* infected animals, macroscopic examination showed luminal distension, erythema and intestinal edema, with the intestinal changes being most pronounced in the cecum and colon, decreasing towards the ileum. All the mice in this group developed a moderate-to-severe typhlocolitis with acute-on-chronic tissue damage that worsened progressively throughout the experiment which resulted in an average cecal and colonic histological injury score of 8 ± 2 and 10 ± 3 (Figure 2(a)) at week 2 and 9 ± 3 and 10 ± 3 (Figure 2(b)) at week 4, respectively (versus noninfected controls *P* < 0.01 for all comparisons). In order to evaluate the efficacy of the treatments in abrogating histological lesions induced by *H. trogontum*, all comparisons were made to the noninfected controls. Macroscopically, no visible abnormalities were found in the cecum and colon of the infected animals that had undergone treatment, while the histological injury score was significantly less in these groups. In the EEN treatment group, the average cecal and colonic scores were 6 ± 4 and 7 ± 3 (Figure 2(a)) at weeks 2 and 3 ± 2 and 5 ± 2 (Figure 2(b)) at week 4, respectively (*P* = NS for all comparisons). In the MNZ group, the average cecal and colonic histological injury scores were correspondingly 6 ± 3 and 6 ± 4 (Figure 2(a)) at week 2 and 5 ± 2 and 5 ± 2 (Figure 2(b)) at week 4 (*P* = NS for all comparisons), while for the EEN + MNZ treated mice the scores were 7 ± 2 and 8 ± 3 (*P* < 0.01 for both comparisons; Figure 2(a)) at week 2and 6 ± 2 and 6 ± 3 (*P* < 0.05 for both comparisons; Figure 2(b)) at week 4. In the HC treated animals, the total cecal and colonic histological injury scores were 8 ± 4 and 8 ± 3 (Figure 2(a)) at week 2 and 9 ± 2 and 9 ± 4 (Figure 2(b)) at week 3, respectively (*P* < 0.01 for all comparisons).

In the noninfected control group, the cecal and colonic mucosal thicknesses were 146 ± 18 *μ*m and 187 ± 16 *μ*m, respectively. The mucosal thickness in the infected controls was significantly higher as compared to the noninfected controls (week 2: cecum 346 ± 7 *μ*m, colon 452 ± 3 *μ*m; week 4: cecum 356 ± 6 *μ*m, colon 454 ± 8 *μ*m; *P* < 0.01 for all comparisons). Except for the mice treated with HC, no significant statistical difference was found in the mucosal thickness of infected but treated mice as compared with the noninfected controls (*P* = NS for all comparisons). The group of mice treated with HC had a slightly lower mean mucosal thickness as compared with the infected controls (week 2: cecum: 320 ± 5 *μ*m, colon 416 ± 6 *μ*m; week 3: cecum 352 ± 9 *μ*m, colon 432 ± 3 *μ*m; *P* = NS for all comparisons), while this was significantly higher than that of the noninfected controls (weeks 2 and 3: *P* < 0.01 for all comparisons).

### 3.3. Epithelial Electrophysiology of Intestinal Mucosa

**Figure 3:**
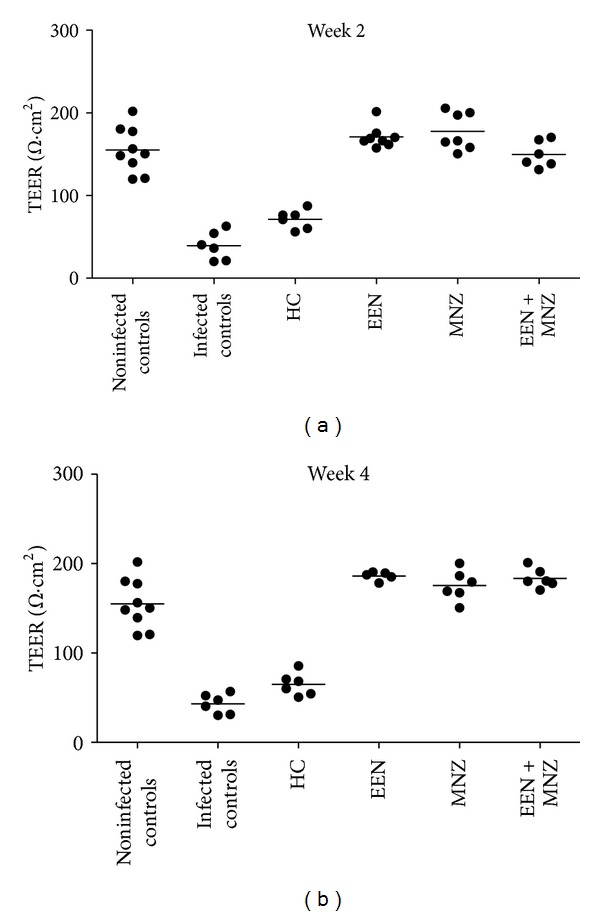
TEER of intestinal mucosa of male and female C57BL/6 mice. Mice from each group were euthanized either 2 weeks (a) or 4 weeks (3 weeks in HC group) (b) following treatment, and colon tissue was collected. Tissue segments were opened along the mesenteric border and then cut into flat sheet and placed into the Ussing chamber with TEER measured. *H. trogontum* significantly decreased TEER in the infected controls (weeks 2 and 4: versus noninfected controls *P* < 0.01 for both comparisons). In contrast, the EEN, MNZ, and EEN + MNZ treatments remarkably ameliorated the increased TEER observed in infected controls (weeks 2 and 4: versus noninfected controls *P* = NS for all comparisons). The group of mice treated with HC had TEER levels below the noninfected controls (weeks 2 and 3: *P* < 0.01 for both comparisons) but elevated slightly but not significantly compared with the infected controls (week 2 and 3: *P* = NS for both comparisons). Bars indicate mean. NS: not significant; *P* > 0.05. TEER: transepithelial electrical resistance; HC: hydrocortisone; EEN: exclusive enteral nutrition; MNZ: metronidazole.

Epithelial electrophysiology Ussing chamber studies were employed to assess gut barrier function in the mice. TEER was calculated to provide an overall assessment of gut barrier functions. TEER was 155 ± 28 Ω·cm^2^ for the noninfected controls and was significantly lower in infected controls (weeks 2 and 4: versus noninfected controls *P* < 0.01 for both comparisons; Figures 3(a) and 3(b)). Interestingly, there was no significant difference in TEER of infected but EEN, MNZ and EEN + MNZ treated mice compared to the noninfected controls (weeks 2 and 4: *P* = NS for all comparisons; Figures 3(a) and 3(b)). The group of mice treated with HC had TEER levels below the noninfected controls (weeks 2 and 3: *P* < 0.01 for both comparisons) but elevated slightly but not significantly compared with the infected controls (weeks 2 and 3: *P* = NS for both comparisons; Figures 3(a) and 3(b)).

**Figure 4:**
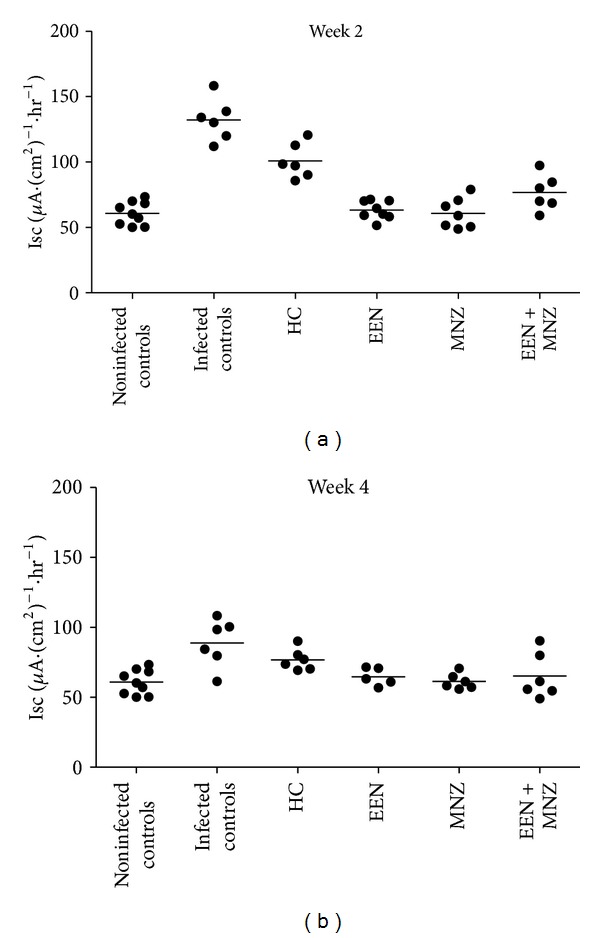
Mucosal tissue Isc measurements in *ex vivo* experiments. Tissue segments were mounted in the Ussing chamber, with Isc measured. (a) Isc levels following 2-week treatment or no treatment (noninfected controls); (b) Isc levels following 4-week treatment (or 3-week treatment for the HC group) or no treatment. In comparison with the noninfected controls, *H. trogontum* induced a significant increase in Isc in the infected controls (weeks 2 and 4: *P* < 0.01 for both comparisons). However, no change in Isc was observed in the infected but (EEN, MNZ and EEN + MNZ) treated mice (weeks 2 and 4: versus noninfected controls *P* = NS for all comparisons). The group of mice treated with HC for either 2 or 3 weeks had reduced Isc levels compared to the infected controls (week 2: *P* < 0.01; week 3: *P* = NS) but were elevated compared to the noninfected control mice (weeks 2 and 3 *P* < 0.01 for both comparisons). Bars indicate mean. NS: not significant; *P* > 0.05. Isc: short-circuit current; HC: hydrocortisone; EEN: exclusive enteral nutrition; MNZ: metronidazole.

Isc was measured to assess ion movement across the epithelium (Figures 4(a) and 4(b)). Isc increased in infected controls compared to noninfected controls (week 2 and 4: *P* < 0.01 for both comparisons; Figures 4(a) and 4(b)). Isc levels in treated mice (EEN, MNZ, and EEN + MNZ) were equivalent to those of the noninfected controls (week 2 and 4: *P* = NS for all comparisons) except for mice treated with HC, where Isc levels were below infected controls (week 2: *P* < 0.01; week 3: *P* = NS) but elevated as compared with the noninfected controls (week 2 and 3: *P* < 0.01 for both comparisons; Figures 4(a) and 4(b)).

### 3.4. Intestinal Tight Junction Permeability to HRP

Macromolecular permeability of the epithelium was assessed by mucosal-to-serosal flux of HRP. A HRP flux of 102 ± 9 nM/hr was calculated for the noninfected controls (Table 2). Consistent with the epithelial electrophysiology results, mucosal-to-serosal HRP flux was significantly increased in infected controls (weeks 2 and 4: versus noninfected controls *P* < 0.01 for both comparisons; Table 2) but unchanged in the infected and EEN, MNZ, EEN + MNZ treated mice (Table 2). Similar to previous findings, the infected but HC treated mice had a HRP flux that was lower than infected controls but still significantly higher than the noninfected controls (Table 2).

### 3.5. Plasma Levels of TNF-*α* and MPO

**Figure 5:**
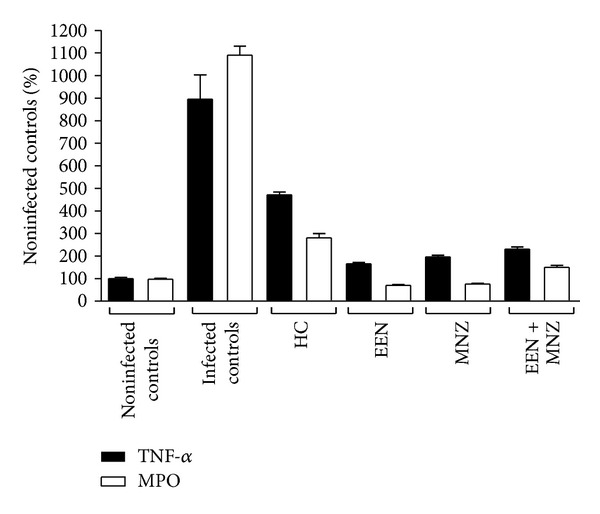
Plasma levels of TNF-*α* and MPO in male and female C57BL/6 mice. At necropsy, following 4-week treatments cardiac puncture was performed to collect blood. The plasma TNF-*α* and MPO activity were then determined using corresponding ELISA kits. *H. trogontum* infected mice (infected controls) displayed a trend of increased plasma TNF-*α* and MPO activity over the 4-week experimental period as compared with the noninfected controls (*P* < 0.01). In contrast, in comparison to the infected controls, the C57BL/6 mice showed significantly lower TNF-*α* and MPO plasma levels in response to the EEN, MNZ, and a combination of EEN and MNZ over the period of 4 weeks (*P* < 0.01 for all comparisons; versus noninfected controls *P* = NS for all comparisons). In the HC group, by week 3, the TNF-*α* and MPO plasma levels were significantly below infected controls (*P* < 0.01 for both comparisons) but remained elevated relative to the noninfected controls (*P* < 0.01 for both comparisons). Data are expressed as percentage of the noninfected controls over the period of 4 weeks. Bars indicate mean. NS: not significant; *P* > 0.05. HC: hydrocortisone; EEN: exclusive enteral nutrition; ELISA: enzyme-linked immunosorbent assay; MNZ: metronidazole.

Next, we examined whether PF and the nonnutritional therapeutic agents used in the current study had the capacity to restore normal inflammatory marker levels in infected animals. As shown in Figure 5, a marked increase in both TNF-*α* and MPO plasma concentrations was observed in the infected mice after *H. trogontum* challenge over the period of 4 weeks (versus noninfected controls *P* < 0.01 for both comparisons). Of particular note, all the treatments, except for HC, were able to eliminate inflammation by reducing TNF-*α* and MPO levels relative to the *H. trogontum* infected controls (*P* < 0.01 for all comparisons; versus noninfected controls *P* = NS for all comparisons; Figure 5). In the HC group, by week 3, both TNF-*α* and MPO plasma levels were also significantly reduced (versus infected controls *P* < 0.01 for both comparisons); however, these levels remained elevated relative to the noninfected controls (*P* < 0.01 for both comparisons; Figure 5).

### 3.6. Intestinal Gene Expression of MIP-2, LIX, and TNF-*α*

As mentioned earlier, the chemokines MIP-2 and LIX (homologs of human IL-8) have been found to be expressed in mouse intestinal epithelial cells and to contribute to the pathology of a number of animal models of disease [59]. Therefore, in the current study, the mucosal inflammatory response was assessed by measuring gene expression of MIP-2, LIX, and TNF-*α*.

**Figure 6:**
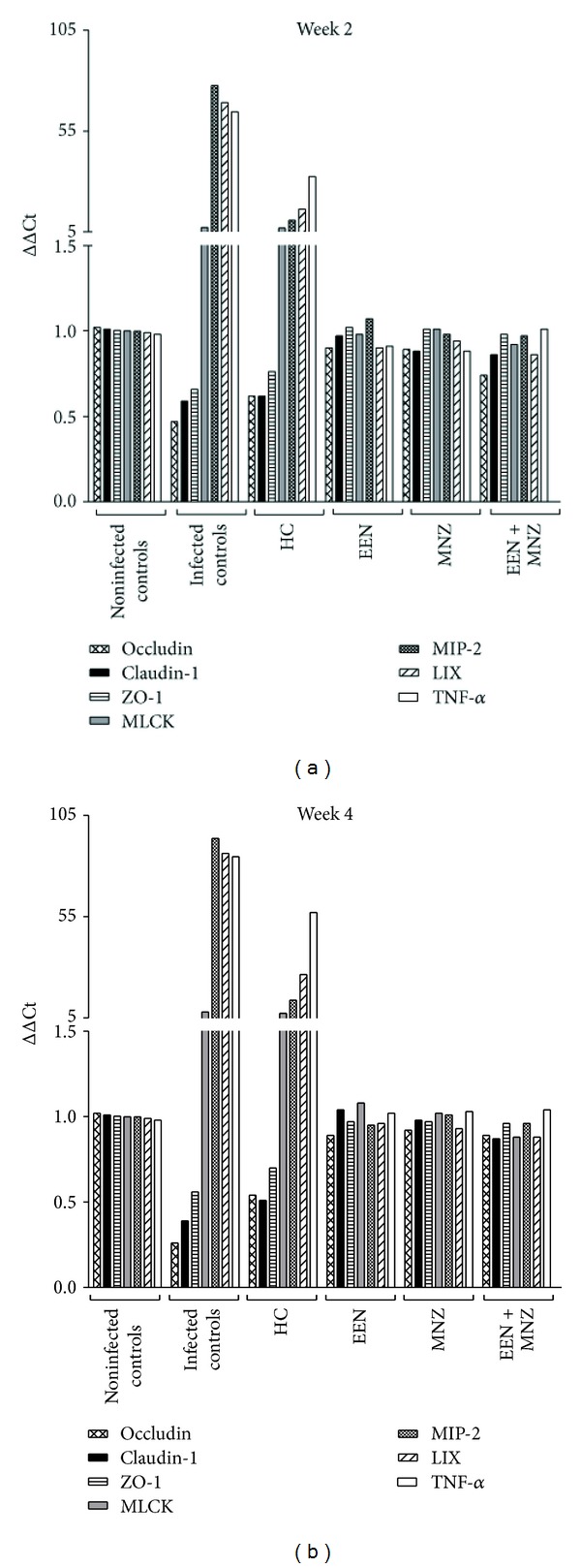
Mucosal gene expression of tight junction structural proteins, MIP-2, LIX, TNF-*α*, and MLCK in male and female C57BL/6 mice. RNA from the colon of each mouse was extracted and subjected to RT-PCR. (a) Gene expression levels following 2-week treatment or no treatment (noninfected controls); (b) gene expression levels following 4-week treatment (or 3-week treatment for the HC group) or no treatment. mRNA expression of occludin, claudin-1 and ZO-1 in the infected control mice were significantly decreased after infection with *H. trogontum* (weeks 2 and 4: versus noninfected controls *P* < 0.01 for all comparisons), while MIP-2, LIX, TNF-*α*, and MLCK gene expressions were upregulated (weeks 2 and 4: versus noninfected controls *P* < 0.01 for all the comparisons). Administration of EEN, MNZ, EEN + MNZ to the infected mice abrogated any effects of *H. trogontum* as mRNA expression levels of genes associated with tight junctions, MIP-2, LIX, TNF-*α*, and MLCK were similar to that of the noninfected controls (week 2 and 4: *P* = NS and versus infected controls *P* < 0.01 for all the comparisons). However, HC only partially downregulated transcription of MIP-2, LIX, and TNF-*α* (weeks 2 and 3: versus infected controls *P* < 0.01 for all comparisons) with a slight rise in gene expression of occludin, claudin-1, and ZO-1 (weeks 2 and 3: versus infected controls *P* = NS for all comparisons). In addition, HC failed to decrease the gene expression levels of MLCK to those of the noninfected controls, even after 3 weeks (weeks 2 and 3: versus infected controls *P* = NS for both comparisons). NS: not significant; *P* > 0.05. MIP-2: macrophage inflammatory protein 2; LIX: lipopolysaccharide-induced CXC chemokine; TNF-*α*: tumor necrosis factor *α*; MLCK: myosin light chain kinase; ZO: zonula occludens; IL-8: interleukin-8; Ct: cycle threshold; HC: hydrocortisone; EEN: exclusive enteral nutrition; MNZ: metronidazole.

In comparison with the noninfected controls, MIP-2, LIX, and TNF-*α* mRNA levels were significantly upregulated in infected controls (weeks 2 and 4: *P* < 0.01 for all comparisons; Figures 6(a) and 6(b)). In mice treated with EEN, MNZ, or EEN + MNZ, there was no difference in MIP-2, LIX, and TNF-*α* mRNA expression, as compared with the noninfected controls (weeks 2 and 4: *P* = NS for all comparisons; Figures 6(a) and 6(b)). In contrast, infected animals treated with HC had MIP-2, LIX, and TNF-*α* expression levels below the infected controls (week 2 and week 3: *P* < 0.01 for all comparisons; Figures 6(a) and 6(b)) but elevated compared with the noninfected controls (weeks 2 and 3: *P* < 0.01 for all comparisons; Figures 6(a) and 6(b)). In addition, mucosal MIP-2, LIX, and TNF-*α* mRNA levels correlated with mucosal-to-serosal HRP flux (Table 3).

### 3.7. Analysis of Tight Junction Proteins and MLCK

Changes in expression of MLCK and tight junction associated genes in response to inflammatory mediators [1, 3, 45] have been reported to play a vital role in epithelial barrier function. To investigate this MLCK, occludin, claudin-1, and ZO-1 gene expressions were assessed. In infected mice, MLCK gene expression was upregulated (weeks 2 and 4: versus noninfected controls *P* < 0.01 for both comparisons); while the gene expression levels of occludin, claudin-1, and ZO-1 were downregulated (weeks 2 and 4: versus noninfected controls *P* < 0.01 for all comparisons; Figures 6(a) and 6(b)). In contrast, mRNA expression levels of genes associated with tight junctions as well as MLCK were not significantly different in the infected but treated mice (weeks 2 and 4: versus noninfected controls *P* = NS for all comparisons; Figures 6(a) and 6(b)). The only exception to this was with HC, where a slight rise in the expression of occludin, claudin-1, and ZO-1 was noted; however, the differences were not significant (weeks 2 and 3: versus infected controls *P* = NS for all comparisons; Figures 6(a) and 6(b)). In this group, gene expression of MLCK did fall slightly but not significantly as compared with the infected control group (weeks 2 and 3: *P* = NS for both comparisons; Figures 6(a) and 6(b)). In addition, mucosal MLCK gene expression correlated closely with mucosal MIP-2, LIX, and TNF-*α* mRNA expression (Table 3).

**Figure 7:**
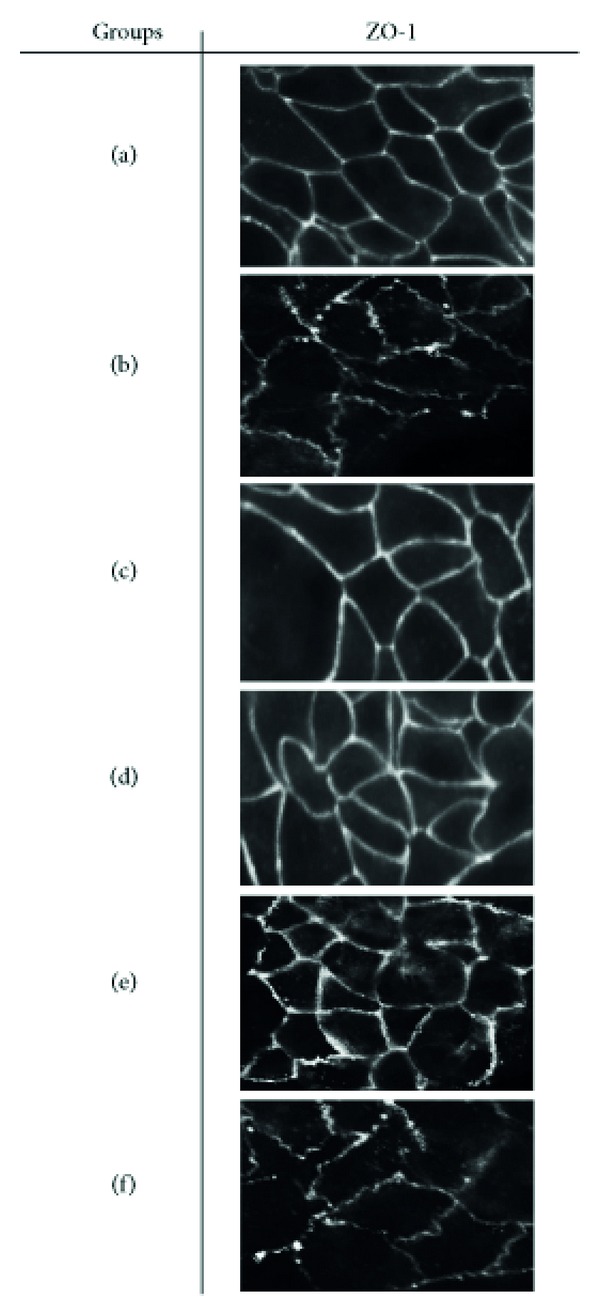
Distribution of ZO-1 in C57BL/6 mice intestinal epithelium. Colon sections from infected/treated and noninfected control groups were fluorescent labelled with antibody to the tight junction protein ZO-1 and were imaged using an Axioplan 2 microscope (Zeiss; ×40 magnification) and AxioVision software (Zeiss). In the noninfected controls (a), the ZO-1 was localized to both the apical lateral borders, as well as between the epithelial cells. In the infected controls (b), however, *H. trogontum* infection disrupted the distribution of the ZO-1 with visible breakage in the ZO-1 strands. *H. trogontum* also induced the ZO-1 internalization with a decrease in staining intensity at the epithelial cell borders (b). Following 4-week treatment with the EEN (c), MNZ (d), and a combination of EEN and MNZ (e), ZO-1 strands appeared intact and were sharply localized to the cellular margins. The breakage in the ZO-1 strands and its displacement in the cell borders observed in the infected controls were not markedly changed by the HC treatment (f). ZO: zonula occludens; HC: hydrocortisone; EEN: exclusive enteral nutrition; MNZ: metronidazole.

ZO-1 cellular localization was also investigated. In the noninfected control animals ZO-1 appeared as a continuous band localized at the intracellular border (Figure 7(a)). For mice infected with *H. trogontum* there appeared a progressive reorganization of the ZO-1 with displacement of the protein away from the cellular border and increased frequency of strand breaks (Figure 7(b)). In infected but (EEN, MNZ, and EEN + MNZ) treated mice, the ZO-1 protein appeared to be restricted to the tight junctions and the cellular periphery similar to the noninfected controls (Figures 7(c)–7(e)). ZO-1 staining in mice treated with HC appeared similar to infected control mice (Figure 7(f)).

## 4. Discussion

Breakdown of the intestinal epithelial barrier in association with luminal bacteria plays an important role in the pathogenesis of IBD [1, 8]. This study examined the effect of several current CD treatments, including HC, EEN, MNZ, and a combination of EEN and MNZ, on the intestinal barrier using an *H. trogontum* infected IL-10 gene-deficient mouse model of colitis. As previously reported by Whary et al. [10], we observed that infection of IL-10 gene-deficient mice with *H. trogontum* induces severe typhlocolitis with tissue damage and disruption of intestinal barrier function. Using this model we observed that treatment with EEN or MNZ abrogated intestinal barrier dysfunction, whereas treatment with HC only partially did. 

The healthy intestinal barrier is composed of a monolayer of epithelial cells sitting on a specialized extracellular matrix [61–64]. The epithelial cells provide an essential part of the mucosal barrier [61–64]. These cells are involved in both initiate and adaptive immune responses [1, 63] and can control the passage of ions, nutrients, water, and macromolecules across the epithelium [62, 65]. Passage occurs through cell pinocytosis and tight junctions [1, 2]. Tight junctions are highly dynamic, forming a seal between adjacent epithelial cells which excludes luminal bacteria, byproducts, and noxious substances [1, 2]. Tight junctions are composed of transmembrane proteins, including occludin, claudins and junctional adhesion molecules [1, 45, 62] which are linked to the actin and myosin filaments through cytoplasmic plaque proteins, including ZO-1 [1, 45, 62]. The correct and intact association of transmembrane and cytoplasmic proteins is critical for maintaining a functional intestinal barrier. Mucosal damage and loss of intestinal barrier function is a feature of IBD [1, 45, 62, 65].

Strong evidence indicating the importance of the epithelium in gut responses in IBD comes from *in vitro* studies of intestinal epithelial cells [45, 62, 66] and supported by murine models of colitis [8, 10, 14]. Several reports based on *in vitro* analysis indicate that increased intestinal permeability in IBD is closely associated with elevated proinflammatory cytokines, including TNF-*α* and IL-8, and decreased tight junction strand numbers and complexity [1, 45, 66]. Elevated proinflammatory cytokines have been shown to promote the transcription of MLCK [45, 62, 63]. When MLCK is activated, it phosphorylates myosin resulting in disruption of tight junction proteins [45, 62, 65]. In addition, transcription of genes associated with tight junctions is inhibited by the proinflammatory cytokines [45, 62]. These events facilitate the disruption of tight junctions and loss of barrier function, leading to a loss of ions and solutes in the form of leaky flux diarrhea [13, 66] and increased penetration of luminal bacteria, antigens, and potentially harmful toxins into the mucosa [1, 62]. Monitoring of these events using electrophysiological measures showed that EEN and MNZ could completely abrogate any loss in barrier function.

The current goal of IBD therapy is not only to control inflammation but to also promote healing of the bowel. The clinical benefits of current therapies are well documented; however, there are only a limited number of studies that have investigated the effects of therapies on the gut epithelium and how these influence mucosal healing. In the current study, we replicated the therapeutic aspects of human IBD in murine model of colitis to investigate their effects on gut epithelium in a murine model of colitis. This included oral HC, EEN, MNZ, and a combination of EEN and MNZ. Of these therapies, only mice receiving EEN and MNZ had complete resolution of gut barrier function, whereas HC treatment only partially restored gut barrier function. A previous report by Oz et al. [67] used IL-10^−/−^ mice which spontaneously developed colitis when exposed to normal gut flora. Mice were then fed an enteral formula diet either rich or poor in transforming growth factor (TGF)-*β*2. Mice with a diet rich in TGF-*β*2 did not develop prolapse or diarrhea and had lower pathological scores in comparison to mice fed the diet lacking TGF-*β*2 [67]. Consistent with the reduced inflammation, the results of the current study also indicated that EEN and MNZ and their combination downregulated mucosal expression of proinflammatory cytokines as well as MLCK to maintain intestinal tight junction function and structure. The improvement in intestinal barrier function, therefore, likely prevented subsequent infiltration of immune cells and upregulation of cytokine expression in response to enteric antigens in the colon. Although EEN, MNZ, and their combination induced mucosal healing and reduced *H. trogontum* load in the majority of mice (EEN 100%; MNZ 75%; EEN + MNZ 75% reduction), to below the threshold of PCR detection, only EEN showed a significant reduction as compared to the infected controls. This indicated that the outcomes were not solely consequent to antibacterial effects. In addition, HC only partially maintained gut barrier integrity and failed to prevent weight loss. HC had no effect in reducing *H. trogontum* load to below the threshold of PCR detection in all animals assayed. This is not surprising as there is no evidence in the literature supporting the reduction of the pathogenic bacterial load as a potential effect of HC. In contrast, several lines of evidence suggest that corticosteroids, including HC, produce their effect on immune cells by activating the glucocorticoid receptors to regulate not only the transcription of various steroid-response target genes involved in inflammatory process but also inhibit the production of a large number of proinflammatory cytokines (e.g., TNF-*α*, IL-1, -6, and -8) [37]. Consistent with this evidence, we ascertained that HC acts primarily by immunosuppressive mechanisms as the animals receiving HC had partial reductions in inflammatory markers (e.g., TNF-*α*, MIP-2, LIX, and MPO). One explanation of HC activity in this model may be that HC does not remove the intestinal proinflammatory bacteria, the anti-inflammatory properties of HC may not be sufficient to completely inhibit inflammation, and therefore the balance swings to continued, albeit a reduced, inflammatory response.

Based on the outcomes of this study, our previous findings [42, 68], and with the help of clinical data [68–70], it is rational to suggest that EEN may act with a similar mechanism(s) to MNZ to induce mucosal healing. There is evidence that both EEN and MNZ have anti-inflammatory properties [22, 30–36, 45, 70]. For instance, de Jong et al. [42] reported that PF has direct anti-inflammatory effects on enterocytes, preventing their chemokine response to proinflammatory mediators in the colonic epithelial cell lines. Several reports have also demonstrated that antibiotics/probiotics directly or indirectly suppress the intestinal immune response, improve mucosal barrier function, and reduce gut translocation of bacteria [30, 32–36, 71–73]. Consistent with this evidence, we ascertained that only groups receiving EEN, MNZ, or a combination of EEN and MNZ had reductions in *H. trogontum* load as well as MIP-2, LIX, and TNF-*α* (key proinflammatory immunological mediators that contribute to tissue injury in the pathogenesis of the disease) mRNA levels in the colonic mucosa. Although *H. trogontum* was still detected in some MNZ mice, in these mice inflammation had resolved, suggesting that MNZ does possess anti-inflammatory properties as well as antibacterial properties. It has been reported that infliximab treatment, which completely blocks inflammation, not only promotes mucosal healing but also reduces bacterial load within the gut [74, 75]. Given this, one might hypothesize that similar results of mucosal healing could be achieved with partial suppression of inflammation plus reduction of bacterial load.

In summary, this study has demonstrated that *H. trogontum* infection in IL-10^−/−^ mice generates acute-on-chronic typhlocolitis, thus reconfirming that infection of IL-10^−/−^ mice with *H. trogontum* produces a picture similar to that observed in IBD, particularly CD. This model could also be considered as a rapid screening tool to evaluate mucosal healing and gut function in response to nutritional and nonnutritional therapies. The current work also ascertained that administration of EEN, MNZ (or a combination of EEN and MNZ) to infected mice reduced mucosal cytokine levels and lead to recovery of barrier function, along with resolution of mucosal inflammatory events and reduction in *H. trogontum* load. The resolution of gut barrier function likely enhanced resolution of the inflammatory process in the mucosa. Whereas with HC treatment, inflammation is reduced even though gut function is not restored which likely inhibits resolution of the inflammatory process. Therefore, these findings provide a plausible explanation as to the observation that CD patients achieve mucosal healing more readily following EEN than following treatment with corticosteroids [23]. While the mechanisms of action of PF are likely to be due to both direct intracellular mechanism(s) and alteration of the gut mucosa, further investigations that will more precisely define these mechanism(s) are required.

## Supplementary Material

Composition of Osmolite (Abbott Nutrition; per 100 mL), as used for polymeric formula (PF) in the current study, is provided in Supplementary Table 1. As mentioned in the materials and methods, all animals, except for Exclusive Enteral Nutrition (EEN) group, were fed standard chow which its chemical composition is summarized in Supplementary Table 2. Cecum and colon tissues, obtained from each mouse at necroscopy, were examined and assigned a histological injury score corresponding to the severity of inflammation observed using a scheme modified from Madsen et al [9]. Histological scores ranged from 0 (minimal injury) to 15 (maximal injury) by scoring 4 criteria including epithelial hyperplasia (0-3), lamina propria mononuclear infiltration (0-4), Neutrophil infiltration (0-4) and mucosal ulceration (0-4) as outlined in Supplementary Table 3. Following histological examination, association of the average histological injury score with the presence and/or absence of *Helicobacter trogontum* in colon tissue, obtained from Interleukin (IL)-10-deficient mice at week 2 and 4, were assessed and the results are summarized in Supplementary Table 4. As shown in Supplementary Figure 1, the presence and/or absence of *H. trogontum* in the colon of noninfected, infected and infected but treated mice was evaluated at week 2 and week 4 using *H. trogontum* 16S rRNA specific polymerase chain reaction (PCR).

## Figures and Tables

**Table 1:** Population, sex, and group description of IL-10^−/−^ mice on a C57BL/6 background.

Group no.	Group description	Total number of mice	
1	Noninfeced control group; no infection and no treatment (66.7% female)	9 (killed time 0)	

Treatments		week 2	week 4

2	Infected control group; treatment: sterilized water (*n* = 12; 58.3% female)	6	6
3	^ 1^HC group; treatment: HC (*n* = 12; 41.7% female)	6	^ 2^6
4	^ 3^EEN group; treatment: ^4^PF (*n* = 13; 46.2% female)	8	5
5	^ 5^MNZ group; treatment: MNZ (*n* = 13; 46.2% female)	7	6
6	EEN + MNZ group; treatment: a combination of PF and MNZ (*n* = 12. 50% female)	6	6

^1^Hydrocortisone; ^2^mice in this group were euthanized at week 3 because of severe diarrhea, dehydration, and weight loss (their indexes were compared to those of other groups at week 4 throughout this study); ^3^exclusive enteral nutrition; ^4^polymeric formula; ^5^metronidazole.

**Table 2:** Mucosal-to-serosal ^1^HRP permeability flux.

Groups	Week 2			Week 4		
	HRP flux (nM/hr)	^2^ *P*	^3^ *P*	HRP flux (nM/hr)	^2^ *P*	^3^ *P*
Infected controls	**150 ± 9**	**<0.01**	—	**141 ± 17**	**<0.01**	—
^ 4^HC	**120 ± 9**	**<0.01**	**<0.05**	**118 ± 5**	**<0.01**	**<0.01**
^ 5^EEN	89 ± 8	NS	**<0.01**	90 ± 10	NS	**<0.01**
^ 6^MNZ	99 ± 3	NS	**<0.05**	85 ± 11	NS	**<0.01**
EEN + MNZ	87 ± 6	NS	**<0.01**	86 ± 10	NS	**<0.01**

^1^Horseradish peroxidase.

Data are mean values ± SD.

^2^ *P* versus noninfeced controls (HRP flux: 102 ± 9 nM/hr).

^3^ *P* versus infected controls.

Significant *P* values are in bold (*P* ≤ 0.05).

NS: not significant; *P* > 0.05.

^4^Hydrocortisone; ^5^exclusive enteral nutrition; ^6^metronidazole.

**Table 3:** Spearman's correlation values for markers of inflammation with ^1^HRP flux and with mucosal MLCK gene expression.

Correlation parameters	^ 2^MIP-2	^ 3^LIX	^ 4^TNF-*α*
HRP	Noninfeced controls: *r* = 0.61, *P* = 0.03	Noninfeced controls: *r* = 0.78, *P* < 0.01	Noninfeced controls: *r* = 0.74, *P* < 0.01
	Infected controls: *r* = 0.68, *P* < 0.01	Infected controls: *r* = 0.71, *P* < 0.01	Infected controls: *r* = 0.75, *P* < 0.01
	^ 5^HC: *r* = 81, *P* < 0.01	HC: *r* = 0.52, *P* = 0.03	HC: *r* = 0.77, *P* < 0.01
	^ 6^EEN: *r* = 0.80, *P* < 0.01	EEN: *r* = 0.53, *P* = 0.04	EEN: *r* = 0.88, *P* < 0.01
	^ 7^MNZ: *r* = 0.72, *P* < 0.01	MNZ: *r* = 0.68, *P* = 0.01	MNZ: *r* = 0.69, *P* < 0.01
	EEN + MNZ: *r* = 0.71, *P* < 0.01	EEN + MNZ: *r* = 0.84, *P* < 0.01	EEN + MNZ: *r* = 0.63, *P* < 0.01

^ 8^MLCK	Noninfeced controls: *r* = 0.82, *P* < 0.01	Noninfeced controls: *r* = 0.69, *P* < 0.01	Noninfeced controls: *r* = 0.79, *P* < 0.01
	Infected controls: *r* = 0.73, *P* < 0.01	Infected controls: *r* = 0.66, *P* = 0.01	Infected controls: *r* = 85, *P* < 0.01
	HC: *r* = 0.67,*P* = 0.01	HC: *r* = 0.84, *P* < 0.01	HC: *r* = 0.76, *P* < 0.01
	EEN: *r* = 0.75, *P* < 0.01	EEN: *r* = 0.64, *P* = 0.02	EEN: *r* = 0.78, *P* < 0.01
	MNZ: *r* = 0.59, *P* = 0.01	MNZ: *r* = 0.77, *P* < 0.01	MNZ: *r* = 0.63, *P* = 0.01
	EEN + MNZ: *r* = 0.68, *P* < 0.01	EEN + MNZ: *r* = 0.71, *P* < 0.01	EEN + MNZ: *r* = 0.69, *P* < 0.01

^1^Horseradish peroxidase;^ 2^macrophage inflammatory protein 2; ^3^lipopolysaccharide-induced CXC chemokine;^ 4^tumor necrosis factor *α*;^ 5^hydrocortisone; ^6^exclusive enteral nutrition; ^7^Metronidazole. ^8^myosin light chain kinase.
