# Supplementary material for: Inflammatory Bowel Disease Therapies and Gut Function in a Colitis Mouse Model
Source: Biomed Res Int. 2013 Aug 6;2013:909613. doi: 10.1155/2013/909613 (PMC3763566; doi:10.1155/2013/909613)
Supplement: Supplementary file 1 — Composition of Osmolite (Abbott Nutrition; per 100 mL), as used for polymeric formula (PF) in the current study, is provided in Supplementary Table 1. As mentioned in the materials and methods, all animals, except for Exclusive Enteral Nutrition (EEN) group, were fed standard chow which its chemical composition is summarized in Supplementary Table 2. Cecum and colon tissues, obtained from each mouse at necroscopy, were examined and assigned a histological injury score corresponding to the severity of inflammation observed using a scheme modified from Madsen et al [9]. Histological scores ranged from 0 (minimal injury) to 15 (maximal injury) by scoring 4 criteria including epithelial hyperplasia (0-3), lamina propria mononuclear infiltration (0-4), Neutrophil infiltration (0-4) and mucosal ulceration (0-4) as outlined in Supplementary Table 3. Following histological examination, association of the average histological injury score with the presence and/or absence of Helicobacter trogontum in colon tissue, obtained from Interleukin (IL)-10-deficient mice at week 2 and 4, were assessed and the results are summarized in Supplementary Table 4. As shown in Supplementary Figure 1, the presence and/or absence of H. trogontum in the colon of noninfected, infected and infected but treated mice was evaluated at week 2 and week 4 using H. trogontum 16S rRNA specific polymerase chain reaction (PCR). [file 909613.f1.pdf]

SUPPLEMENTARY TABLES AND FIGURE LEGENDS

Supplementary Table 1. Composition of Osmolite

(Abbott Nutrition, Australia Pty Ltd, NSW)

		Per 100 ml
Energy	(KJ)	1061
	(KCal)	252
Protein (g)		4.00
Carbohydrate (g)		13.6
of which sugar (g)		0.63
Fat (g)		3.40
of which saturates (g)		0.84
Fibre (g)		0
	Vitamins	
Vitamin A (RE; µg)		108
Vitamin D (µg)		0.73
Vitamin E (αTE; mg)		2.14
Vitamin C (mg)		10.0
Thiamine (vitamin B1; mg)		0.16
Riboflavin (vitamin B2; mg)		0.18
Niacin (NE; mg)		1.7
Vitamin B6 (mg)		0.22
Folacin (folic acid; µg)		23.0
Vitamin B12 (µg)		0.34
Biotin (µg)		4.6
Pantothenic acid (mg)		0.78
Vitamin K (µg)		5.2
	Minerals	
Sodium (mg)		88.0
Calcium (mg)		68.0
Phosphorus (mg)		68.0
Iron (mg)		1.40
Magnesium (mg)		20.0
Zinc (mg)		1.30
Iodine (µg)		11.0
Potassium (mg)		148
Chloride (mg)		136
Copper (µg)		170
Manganese (mg)		0.38
Selenium (µg)		6
Chromium (µg)		6.5
Molybdenum (µg)		12.0
Choline (mg)		56.0

- Osmolarity 244 mOsm/L

Supplementary Table 2. Chemical composition of the standard mouse chow diet (Lab Diet 5001)

Nutrients	
**Proteins, %**	**23.9**
Arginine,%	1.41
Cystine, %	0.31
Glycine, %	1.21
Histidine, %	0.57
Isoleucine, %	1.14
Leucine, %	1.83
Lysine, %	1.41
Methionine, %	0.67
Phenylalanine, %	1.04
Tyrosine, %	0.71
Threonine, %	0.91
Tryptophan, %	0.29
Valine, %	1.17
Serine, %	1.19
Aspartic Acid, %	2.81
Glutamic Acid, %	4.37
Alanine, %	1.43
Proline, %	1.49
Taurine, %	0.02
**Fat (ether extract), %**	**5.00**
**Fat (acid hydrolysis), %**	**5.70**
Cholesterol, ppm	200
Linoleic Acid, %	1.22
Linolenic Acid, %	0.10
Arachidonic Acid, %	<0.01
Omega-3 Fatty Acids, %	0.19
Total Saturated Fatty Acids, %	1.56
Total Monounsaturated Fatty Acids, %	1.60
**Fiber (Crude), %**	**5.10**
Neutral Detergent Fiber, %	15.6
Acid Detergent Fiber, %	6.70
**Nitrogen-Free Extract (by difference), %**	**48.7**
Starch, %	31.9
Glucose, %	0.22
Fructose, %	0.30
Sucrose, %	3.70
Lactose, %	2.01
**Total digestible nutrients, %**	**76.0**
**Gross Energy, kcal/g**	**4.07**
**Physiological Fuel Value, kcal/g**	**3.36**
**Metabolizable Energy, kcal/g**	**3.02**
**Minerals**	
**Ash, %**	**7.00**
Calcium, %	0.95

Phosphorus, %	0.66
Phosphorus (non-phytate), %	0.39
Potassium, %	1.18
Magnesium, %	0.21
Sulfur, %	0.36
Sodium, %	0.40
Choline, %	0.67
Fluorine, ppm	16.00
Iron, ppm	270
Zinc, ppm	79.00
Manganese, ppm	70.00
Copper, ppm	13.00
Cobalt, ppm	0.90
Iodine, ppm	1.00
Chromium, ppm	1.20
Selenium, ppm	0.30
**Vitamins**	
Carotene, ppm	2.30
Vitamin K (as menadione), ppm	1.30
Thiamine Hydrochloride, ppm	16.00
Riboflavin, ppm	4.50
Niacin, ppm	120.00
Pantothenic Acid, ppm	24.00
Choline Chloride, ppm	2250.00
Folic Acid, ppm	7.10
Pyridoxine, ppm	6.00
Biotin, ppm	0.60
B_{12}, mcg/kg	50.00
Vitamin A, IU/g	15.00
Vitamin D (added), IU/g	4.50
Vitamin E, IU/g	42.00

- Oval pellet, 10 mm x 16 mm x 25 mm length

Supplementary Table 3. Histological injury scoring system in IL-10$^{-/-}$ mice on a C57BL/6 background

Histological injury scoring system	Score (0-15)*
**Epithelial hyperplasia**	**0-3**
Normal	0
Mild	1
Moderate	2
Pseudopolyps	3
**Mononuclear infiltration**	**0-4**
None; Rare inflammatory cells in the lamina propria	0
Minimal; very few numbers of mononuclear cells in the lamina propria	1
Moderate; increased numbers of mononuclear cells in the lamina propria	2
Marked; confluence of mononuclear cells extending into sub-mucosa	3
Severe; trans-mural extension of the mononuclear cells infiltration	4
**Neutrophil infiltration**	**0-4**
None; Rare neutrophil cells in the lamina propria	0
Minimal; very few numbers of neutrophil cells in the lamina propria	1
Moderate; increased numbers of neutrophil cells in the lamina propria	2
Marked; confluence of neutrophil cells extending into sub-mucosa	3
Severe; trans-mural extension of neutrophil cells infiltration	4
**Mucosal ulceration**	**0-4**
Normal	0
Surface inflammation	1
Erosions	2
Focal ulceration	3
Multifocal or diffuse ulceration	4

*0-5: normal histological appearance; 6-8: mild histological changes; 9-12: moderate histological changes; 12< severe tissue damage

Supplementary Table 4. Association of the average histological injury score with the presence of *H. trogontum* in colon specimens collected from IL-10$^{-/-}$ mice

Groups	Mouse number		16S rRNA	Average histological injury score*
Non-infected controls	week 2	25	Negative	2
		61	Negative	1
	week 4	26	Negative	1
		62	Negative	1
Infected controls	week 2	1	Negative	11
		30	Positive	14
		47	Positive	12
		70	Positive	11
	week 4	9	Positive	14
		19	Negative	13
		44	Positive	14
		42	Positive	13
[1]HC	week 2	4	Positive	10
		20	Positive	10
		71	Positive	9
		59	Positive	9
	week 4	50	Positive	13
		13	Positive	13
		10	Positive	11
		16	Positive	13
[2]EEN	week 2	23	Negative	5
		2	Negative	4
		53	Negative	3
		48	Negative	4
		72	Negative	5
	week 4	55	Negative	2
		66	Negative	2
		49	Negative	1
[3]MNZ	week 2	29	Negative	4
		5	Negative	4
		63	Positive	8
		64	Negative	2
	week 4	28	Negative	2
		8	Negative	2
		35	Negative	5
		38	Positive	6
EEN+MNZ	week 2	27	Negative	5
		21	Positive	10
		37	Negative	5
		67	Negative	2
	week 4	6	Negative	4
		3	Positive	5
		43	Negative	2
		58	Negative	9

[1]Hydrocortisone; [2]exclusive enteral nutrition; [3]metronidazole

Supplementary Figure 1 legend:

DNA was extracted from colon tissue, which had been stored in *RNAlater*®, and subjected to an *H. trogontum* 16S rRNA specific PCR. Samples displaying a single (888 bp fragment) band were considered PCR positive for *H. trogontum*.

Ladder [fibronectin (FN)-1 marker; Fisher Biotech]; lanes 1-4: non-infected controls; lanes 5-8: infected controls; lanes 9-12 HC; lanes 13-16: MNZ; lanes 17-20: EEN; lanes 21-24: EEN+MNZ

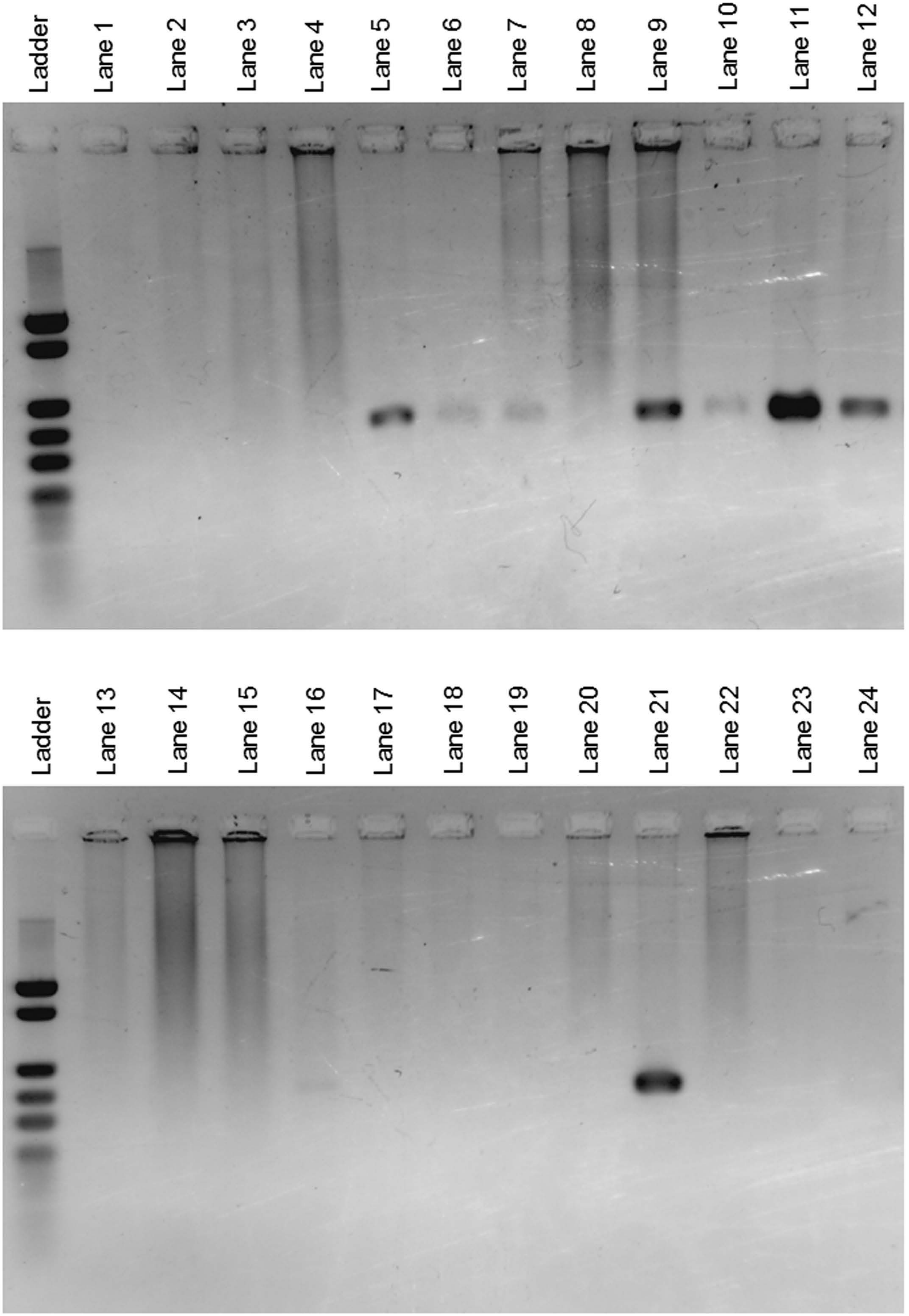
Ladder
Lane 1
Lane 2
Lane 3
Lane 4
Lane 5
Lane 6
Lane 7
Lane 8
Lane 9
Lane 10
Lane 11
Lane 12
Ladder
Lane 13
Lane 14
Lane 15
Lane 16
Lane 17
Lane 18
Lane 19
Lane 20
Lane 21
Lane 22
Lane 23
Lane 24
